# Topical JAK (Janus Kinase) Inhibitor Therapy for Chronic External Otitis in Atopic Dermatitis: A Case Report

**DOI:** 10.7759/cureus.84827

**Published:** 2025-05-26

**Authors:** Kojiro Hirano, Yuni Hirano, Miharu Yu, Yuka Tei, Toshikazu Shimane

**Affiliations:** 1 Department of Otorhinolaryngology - Head and Neck Surgery, Showa Medical University, Tokyo, JPN; 2 Department of Dermatology, Showa Medical University, Tokyo, JPN

**Keywords:** atopic dermatitis (ad), delgocitinib, external ear eczema, interleukin-31, janus kinase (jak) inhibitor, refractory otitis

## Abstract

Atopic dermatitis (AD) often presents challenges in treating external ear lesions and frequently exhibits resistance to conventional therapies. This case report describes a 49-year-old male patient with a history of AD and habitual ear cleaning who developed chronic external otitis that was refractory to topical steroids and steroid ear drops. The patient demonstrated significant improvement following the administration of a topical Janus kinase (JAK) inhibitor, delgocitinib ointment. Delgocitinib effectively suppressed interleukin-31 (IL-31), thereby interrupting the itch-scratch cycle and facilitating the resolution of inflammation. This case suggests that delgocitinib ointment is a promising therapeutic alternative for refractory external ear eczema in patients with AD. Further studies are warranted to evaluate its long-term efficacy and safety in otological applications.

## Introduction

Patients frequently visit otolaryngology clinics with complaints of ear pruritus and otorrhea. Most cases are caused by external ear eczema associated with habitual ear picking or by infectious otitis externa and are typically treated with topical steroids or antibiotic ear drops. However, long-term steroid use carries the risks of skin atrophy and fungal infections [1], while antibiotic resistance remains a significant concern [2]. Recently, biological agents and Janus kinase (JAK) inhibitors have been increasingly used to treat dermatitis [3], improving disease control by suppressing the inflammatory cytokines associated with type 2 inflammation [4]. Biologic agents and JAK inhibitors have demonstrated established efficacy in atopic dermatitis and are widely reported to serve as steroid-sparing treatment options for refractory cases. Interleukin (IL)-4, IL-13, and IL-31 play crucial roles in pruritus; therefore, their inhibition is a key therapeutic strategy [5]. This report presents a case of external ear eczema refractory to topical steroids and dupilumab (an IL-4 and IL-13 receptor antibody), which showed remarkable improvement with delgocitinib ointment, a topical JAK inhibitor.

## Case presentation

A 49-year-old male patient presented with persistent ear pruritus. His medical history included atopic dermatitis, bronchial asthma, hypertension, cataracts, and glaucoma. He had no history of smoking or alcohol consumption. The patient reported multiple allergies, including drug allergies such as a rash induced by theophylline and hand and foot pain associated with upadacitinib, as well as food allergies to banana, rice, wheat, and soy. His medications included oral bilastine, telmisartan, and carbocisteine, along with several topical agents: hydrocortisone butyrate, dimethyl isopropyl azulene, prednisolone valerate acetate, beclometasone propionate, and heparinoids.

Since childhood, the patient had been managed for atopic dermatitis with topical corticosteroids and oral antihistamines. Fourteen months prior to his current presentation, treatment with dupilumab was initiated, resulting in a marked improvement in trunk lesions, with the Eczema Area and Severity Index (EASI) score decreasing from 34.2 to 1.2. Despite this improvement, severe ear pruritus persisted. Treatment with topical steroid ear drops (betamethasone sodium phosphate) and steroid ointments (betamethasone valerate and gentamicin sulfate) proved ineffective, prompting referral to our department.

**Figure 1:**
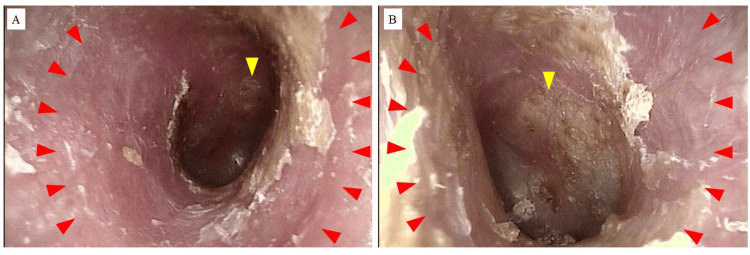
Initial otoscopic findings (A) Findings of the right external auditory canal at the initial visit. A significant amount of scales is adherent to the external auditory canal (red arrowheads), which appears erythematous. Erythema and scales are also observed on the tympanic membrane (yellow arrowhead). (B) Findings of the left external auditory canal at the initial visit. A significant amount of scales is adherent to the external auditory canal (red arrowheads), which appears erythematous. Scales are also observed on the tympanic membrane (yellow arrowhead).

On initial otoscopic examination, erythema and abundant scaling were observed in the external auditory canal (Figure 1).

Laboratory tests showed significant reductions in serum IgE and thymus activation-regulated chemokine (TARC) levels following dupilumab therapy, although both remained above normal ranges (Table 1).

**Table 1:** Laboratory Test Results

Parameters	Value
White Blood Cell Count (WBC)	4,700/μL
Peripheral Blood Eosinophils	12.4% (580/μL)
Immunoglobulin E (IgE)	2,646 IU/mL
Pre-Dupilumab IgE	14,882 IU/mL
Thymus Activation-Regulated Chemokine (TARC)	1,047 pg/dL
Pre-Dupilumab TARC	18,940 pg/dL

Allergy testing demonstrated high sensitization to multiple environmental and food antigens (Table 2).

**Table 2:** Allergen-Specific IgE Levels

Allergen	IgE Level (Ua/mL)	Class
Dust Mites	96.2	5
House Dust	71.1	5
Moths	36.2	4
Molds	12.3	3
Animal Epithelium	15.2	3
Alder	≥100	6
Japanese Cedar	87.5	5
Japanese Cypress	34.3	4
Orchard Grass	30.3	4
Ragweed	45.0	4
Mugwort	38.7	4

Microbiological cultures from the external auditory canal detected only commensal bacteria, with no pathogenic organisms identified (Table 3).

**Table 3:** Bacterial Culture Results of the External Auditory Canal

Bacterial Species	Presence
Staphylococcus aureus	Few
Staphylococcus capitis	Few
*Corynebacterium* species	Few

**Figure 2:**
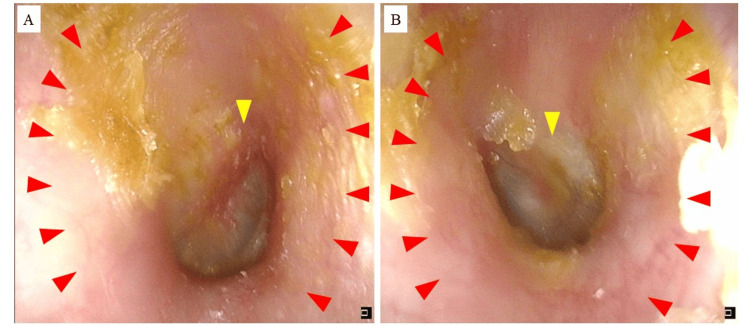
Otoscopic findings, bilaterally observed, after one month (A) Findings of the right external auditory canal after one month of treatment. Erythema of the external auditory canal has improved, and the scaling has subsided (red arrowheads). However, the tympanic membrane remains erythematous (yellow arrowhead). (B) Findings of the left external auditory canal after one month of treatment. Both erythema and scaling of the external auditory canal have subsided (red arrowheads). The tympanic membrane still shows slight erythema (yellow arrowhead).

Due to persistent symptoms despite conventional steroid therapies, we provided instruction on appropriate application techniques for topical ointments and initiated delgocitinib ointment (Corectim® 0.5%). In accordance with the package insert, delgocitinib ointment was applied in small amounts to the external ear twice daily. After one month of treatment, the patient showed substantial improvement, with resolution of scaling and reduced erythema, allowing for the discontinuation of steroid ointment and continuation of delgocitinib alone (Figure 2).

**Figure 3:**
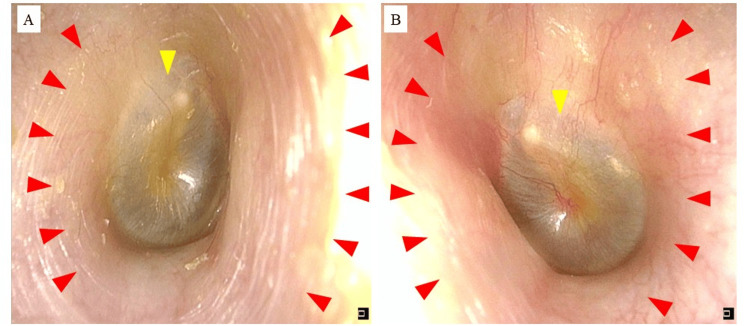
Otoscopic findings, bilaterally observed, after 12 months (A) Findings of the right external auditory canal after one year. Findings are normal in both the external auditory canal (red arrowheads) and the tympanic membrane (yellow arrowhead). (B) Findings of the left external auditory canal after one year. Findings are normal in both the external auditory canal (red arrowheads) and the tympanic membrane (yellow arrowhead).

The patient was subsequently followed up every two months, during which his condition remained stable. Although intermittent use of delgocitinib ointment was prescribed, the patient required only one to two applications per week. Even after one year of intermittent use, remission was sustained without the need for corticosteroids (Figure 3).

## Discussion

Self-ear cleaning habits are common, with 93.4% of the individuals engaging in this practice, 85.1% of whom use cotton swabs [6]. Earwax lubricates, cleans, and protects the external auditory canal. Self-ear cleaning removes the wax, potentially leading to ear infections, trauma, and perforation of the tympanic membrane, as objects are inserted blindly into the ear canal. Wax removal alters the integrity of the ear's natural physiological defenses [6]. Topical steroid therapy remains the mainstay of treatment for chronic otitis externa and is often combined with antibacterial ear drops for bacterial infections and antifungal agents for fungal infections [7]. However, refractory cases are common in patients with habitual ear cleaning, hearing aid or earphone use, atopic dermatitis, seborrheic dermatitis, diabetes, renal failure, allergies, or immunodeficiency [8].

Atopic dermatitis is characterized by an impaired skin barrier function due to filaggrin gene mutations [9]. Maintaining remission requires appropriate self-care. However, lesions in the external auditory canal are particularly challenging to manage and often lead to refractory diseases. Treatment of atopic dermatitis requires interrupting the "itch-scratch cycle" [10], which plays a critical role in disease persistence. To achieve this, appropriate topical steroid therapy is essential. Proactive therapy, involving early suppression of inflammation with topical steroids followed by a gradual reduction in application frequency over more than one month, is recommended to induce and maintain remission [11].

Delgocitinib inhibits all members of the JAK family (JAK1, JAK2, JAK3, and TYK2), thereby suppressing cytokine-induced STAT protein phosphorylation. This mechanism attenuates the activation of immune and inflammatory cells, including T cells, B cells, mast cells, and monocytes, leading to localized suppression of inflammation at the site of application [12]. Additionally, delgocitinib inhibits IL-31-induced pruritus, contributing to symptom relief [13]. In this case, dupilumab administration effectively suppressed the inflammatory cytokines involved in pruritus (IL-4 and IL-13), resulting in well-controlled trunk lesions. However, external ear eczema remained refractory, likely because of persistent habitual ear cleaning. Delgocitinib successfully inhibits IL-31-induced pruritus, contributing to the cessation of habitual ear manipulation. IL-31 is a key factor in the itch-scratch cycle [10,13], and its inhibition is crucial for treating external ear eczema. Topical JAK inhibitors play a pivotal role in suppressing inflammation and maintenance remission.

Additionally, a proactive therapy approach, involving early suppression of acute inflammation followed by a gradual reduction in topical application to sustain remission, was applicable in this case [11]. This strategy facilitated the discontinuation of steroid treatment within one month, with remission maintained using delgocitinib ointment alone. Although topical steroids have broad and potent anti-inflammatory effects, their long-term use is associated with adverse effects, such as skin atrophy and an increased risk of fungal infections [1]. In contrast, JAK inhibitors, while influencing immune responses, have been associated with the potential risks of autoimmune disorders and malignancies [12]. However, as a topical formulation, delgocitinib primarily induces localized side effects such as burning sensation, irritation, rash, pruritus, and dryness [13], with relatively lower systemic risk compared to systemic JAK inhibitors. Despite the high prevalence of external ear eczema, research on its treatment remains limited, and therapeutic options are not yet well established.

## Conclusions

In patients with atopic dermatitis, external ear lesions can become severe, often requiring prolonged steroid therapy. In such cases, delgocitinib ointment may serve as an effective alternative for symptom control, offering a steroid-sparing treatment option for refractory external ear eczema. In the present case, delgocitinib ointment led to a greater improvement in both otoscopic findings and patient satisfaction compared to topical steroids. This effect is likely attributable to delgocitinib's ability to alleviate IL-31-mediated pruritus, thereby enabling the patient to break free from the "itch-scratch cycle."
